# An Optimization Method for an Active Multi-Unit Prosthetic Socket with Dynamic Adaptability in Multi-Task Scenarios

**DOI:** 10.3390/biomimetics11020129

**Published:** 2026-02-11

**Authors:** Yawen Hu, Li Jiang, Chunying Zou, Bangchu Yang, Tianquan Han, Ming Cheng

**Affiliations:** 1 State Key Laboratory of Robotics and System, Harbin Institute of Technology, Harbin 150008, China; huyawen@stu.hit.edu.cn (Y.H.);; 2 Beijing Institute of Space Launch Technology, Beijing 100076, China

**Keywords:** artificial limbs, biomechanical phenomena, optimization, prosthetic interface

## Abstract

As a core functional component of the prosthetic system, the prosthetic socket’s adaptability to the residual limb is directly correlated with the prosthetic’s performance, comfort level, and safety profile. Although traditional sockets can satisfy basic suspension requirements, they commonly suffer from inherent drawbacks in practical applications, including uneven pressure distribution, poor air permeability, and inadequate adaptability to the morphological variations of individual residual limbs. To enhance socket adaptability across multi-task scenarios, this study proposes an intelligent physiological adaptation-based optimal design method for active upper-limb prosthetic sockets. Specifically, this method first employs a dynamic force optimization algorithm for multi-contact units oriented to prosthetic manipulation tasks, which real-timely optimizes the output force of each unit under varying external loads to achieve stable socket suspension with minimal interface pressure. Second, biomechanical experiments are conducted to obtain the pain threshold distribution characteristics of forearm soft tissues under compressive loads, thereby providing a physiological basis for the spatial layout of the contact units. Furthermore, the mechanical performance of different socket structures is evaluated under various representative task scenarios, with peak normal force, mean normal force, and force distribution variance adopted as the key comfort evaluation indices. The results demonstrate that the proposed active multi-unit socket, particularly the double-layered eight-unit symmetric radial staggered configuration, enables a robust balance between comfort and stability across diverse task scenarios, thereby establishing an effective and scalable design paradigm for long-term adaptive upper-limb prosthetic sockets.

## 1. Introduction

The prosthetic socket serves as the critical physical interface connecting the prosthesis to the residual limb. Compared to osseointegration [1,2,3,4], the socket offers advantages including high safety, great applicability, low manufacturing costs, and ease of maintenance, making it the most widely adopted method for human–machine physical connection. Its core functions lie in the secure and reliable transmission of force and motion [5,6], and it provides a bidirectional neural information interface for intelligent prostheses [7]. However, issues such as poor interface conformity between the socket and residual limb, excessive long-term localized pressure, and protracted design cycles directly impact prosthetic functionality, wearer comfort, and patient acceptance of the prosthesis [8].

Traditional fully enclosed sockets employ rigid structures (or those with flexible liners applied to their surfaces) to encase the residual limb [9]. The uniformity of force distribution depends on the congruence between the socket shape and the residual limb’s morphology, where the pressure specifically refers to the normal contact pressure at the socket–skin interface, which is directly related to local soft tissue loading, comfort, and the risk of pressure-induced injury. Early sockets primarily focused on providing basic suspension. For instance, the Muenster full-contact socket achieves stability through comprehensive contact and snug fit with the residual limb. The Northwestern socket incorporates biomechanical principles into its design, reducing restriction on elbow range of motion. To enhance fitting accuracy, current full-contact sockets are typically customized using computer-aided design (CAD) and additive manufacturing (e.g., 3D printing) based on 3D scans or CT images of the residual limb [5]. Nevertheless, the full-contact structure is prone to issues such as inadequate heat dissipation, pressure on sensitive areas, stress concentration, and complex adaptation processes [10,11]. In response, researchers propose frame-type socket designs, which selectively reduce contact area to transfer loads to pressure-resistant regions.

Frame-type sockets demonstrate advantages in improving joint mobility and thermal dissipation [12,13]. For instance, the 3/4-type socket uses an open structure near the elbow to enhance ventilation and range of motion [14]. The adjustable suspension socket proposed by Radocy employs an inner silicone liner to accommodate volume changes of the residual limb during low- and high-intensity activities, enabling dual-mode suspension adjustment [15]. Conventional sockets with static force distributions have significant limitations regarding safety and comfort. Sustained high interface pressure can impede soft tissue blood circulation, increasing the risk of pressure ulcers [16,17,18]. Current research on adjustable sockets predominantly focuses on lower limb prostheses, primarily employing pneumatic mechanisms to accommodate residual limb volume fluctuations or switching ‘squeeze-release’ dual-state [8,19,20,21,22,23].

Lower limb prosthetics must accommodate significant volume changes [24,25]; upper limb prostheses focus more on ensuring precise force transmission, torsional stability, and minimizing pressure on the residual limb [26]. Therefore, the design of upper limb sockets should minimize interface pressure as far as practicable while fulfilling the requirements of operational tasks.

This study aims to enhance the biomechanical compatibility of prosthetic sockets, providing an active and intelligent optimization strategy for the long-term healthy adaptation of upper-limb prosthetic sockets. Firstly, a multi-unit pressure optimization algorithm is proposed to dynamically adjust in real time the minimal force required for each independent control unit to maintain stable suspension of the socket. Secondly, biomechanical experiments identify load-tolerant zones of the forearm soft tissues to optimize spatial layout and enhance biomechanical compatibility. Furthermore, based on the pressure optimization algorithm, the mechanical performance of different unit layouts is evaluated under typical task scenarios to select the configuration with the optimal mechanical characteristics.

The remainder of this paper is organized as follows. Section 2 presents a task-oriented dynamic force optimization algorithm for multi-contact units. Section 3 describes the biomechanical experiments conducted to identify load-tolerant regions of the forearm and evaluates the mechanical performance of different spatial layouts under typical task scenarios. Section 4 discusses the biomechanical implications, practical implementation considerations, and adaptability advantages of the proposed design, followed by the conclusions of this study.

## 2. Dynamic Force Optimization of Multi-Contact Units

**Figure 1:**
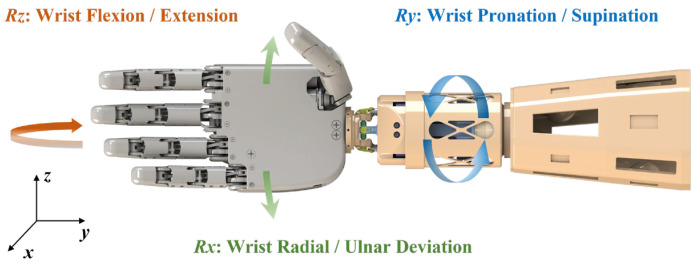
Physical schematic of the upper-limb prosthetic system.

This section proposes a task-oriented dynamic force optimization algorithm for multi-contact units in an upper-limb prosthetic socket. As illustrated in Figure 1, the prosthetic socket operates as a critical interface within the upper-limb prosthetic system, where external loads induced by typical manipulation tasks are transmitted through the prosthesis to the socket–residual limb interface. These task-dependent external forces and moments are dynamically balanced by the coordinated action of multiple contact units. The proposed algorithm dynamically adjusts the output force of each unit according to the external loads experienced by the prosthesis, aiming to achieve stable socket suspension with minimal interface pressure, thereby synergistically enhancing functional adaptability, comfort, and safety.

The socket consists of *n* functionally identical control units, each capable of independently adjusting the normal force applied to the skin surface of the residual limb. This generates a controllable dynamic friction field on the residual limb surface, responding to external loads to prevent relative sliding or rotation at the interface. The objective of pressure optimization is to determine the force distribution at the unit–tissue interface, subject to the following conditions: (1) balance the force vector applied to the prosthetic hand; (2) minimize the output force of the units; and (3) match the mechanical characteristics of the human–machine interface.

The contact force vector applied by the *i*-th unit of the socket is defined as $f_{i}={\left[f_{i,n},\;f_{i,t1},\;f_{i,t2}\right]}^{T}$, where $f_{i,n}\geq 0$ represents the normal force perpendicular to the residual limb skin surface, and $f_{i,t1}$, $f_{i,t2}$ denote orthogonal tangential force components. To prevent slippage between the unit and residual limb, the output of each unit must satisfy the friction constraint given by

(1) $$\sqrt{f_{i,t1}^{2}+f_{i,t2}^{2}}\leq \mu_{i}f_{i,n}$$

where *μ_i_* > 0 denotes the equivalent friction coefficient at the unit–skin interface. In this study, each contact unit is covered with a medical-grade silicone layer to improve comfort and biocompatibility. Accordingly, *μ_i_* is selected as 0.4 based on reported friction coefficients between silicone materials and human forearm skin in the literature [27,28].

Furthermore, the present analysis focuses on quasi-static task scenarios typical of daily upper-limb prosthetic use, in which external loads vary slowly relative to the control update frequency of the active units. Dynamic impact effects are therefore not explicitly modeled. Their influence can be conservatively incorporated into the framework by representing them as increased external load inputs, which are subsequently accommodated through real-time pressure redistribution while maintaining the friction constraints.

External loads applied to the prosthetic system (e.g., self-weight and mass of grasped objects) must be balanced by the forces from all units, as expressed by the force equilibrium equation

(2) $$GF_{unit}=F_{ext}$$

where $F_{unit}={\left[f_{1}^{T},\;f_{2}^{T},\ldots ,\;f_{N}^{T}\right]}^{T}$ denotes the system force vector composed of the output forces from all units; $F_{ext}={\left[F_{x},\;F_{y},\;F_{z},\;M_{x},\;M_{y},\;M_{z}\right]}^{T}$ represents the external load vector, *F_x_*, *F_y_*, *F_z_* denote the external force components along the *x*-, *y*-, and *z*-axes, respectively, and *M_x_*, *M_y_*, *M_z_* denote the corresponding moments about these axes; and $G=\left[G_{1}\;G_{2}\cdot \cdot \cdot G_{n}\right]\in R^{6\times 3n}$ is the force transmission matrix, which maps the unit pressures to the prosthesis center of gravity and depends on the positions of the units. Let $r_{i}={\left[\begin{array}{ccc} lr_{ix} & r_{iy} & r_{iz} \end{array}\right]}^{T}$ be the position vector of the *i*-th unit in the residual limb coordinate system and $x_{i},\;y_{i},\;z_{i}$ be the unit direction vectors corresponding to each coordinate axis in the contact coordinate system. The transformation matrix *G_i_* for each unit is

(3) $$G_{i}=\left[\begin{array}{ccc} z_{i} & x_{i} & y_{i}\\ r_{i}\times z_{i} & r_{i}\times x_{i} & r_{i}\times y_{i} \end{array}\right]$$

The unidirectional force constraint, nonlinear friction constraint, and force equilibrium constraint are equivalently transformed into a positive definiteness condition (*P_i_* > 0) of a descriptive matrix subject to linear constraints. Consequently, without any approximation or simplification, the original nonlinear pressure planning problem is converted into a convex optimization problem on the symmetric positive definite matrix manifold under linear constraints [29,30,31]. The descriptive matrix *P_i_* for each unit is defined as

(4) $$P_{i}=\left[\begin{array}{ccc} \mu_{i}f_{i,n} & 0 & f_{i,t1}\\ 0 & \mu_{i}f_{i,n} & f_{i,t2}\\ f_{i,t1} & f_{i,t2} & \mu_{i}f_{i,n} \end{array}\right]$$

For a socket system comprising *n* units, the global descriptive matrix is the block diagonal matrix $P=\mathrm{diag}\left(P_{1},P_{2},...,P_{n}\right)$.

The force optimization process aims to minimize an objective function defined as

(5) $$\Phi \left(P\right)=\mathrm{tr}\left(W_{p}P\right)+\mathrm{tr}\left(W_{i}P^{-1}\right)$$

where *W_p_* and *W_i_* are diagonal weighting matrices. The first term, tr(*W_p_ P*), enhances comfort by suppressing interfacial pressure through minimizing contact force; the second term, tr(*W_i_P*^−1^), ensures uniformity and safety by penalizing force distribution variance and proximity to friction boundaries. Adjusting the relative weights of *W_p_* and *W_i_* enables dynamic trade-offs between task requirements. For instance, uniformity takes precedence during high-precision operations, whereas pressure suppression is prioritized during prolonged wear.

**Figure 2:**
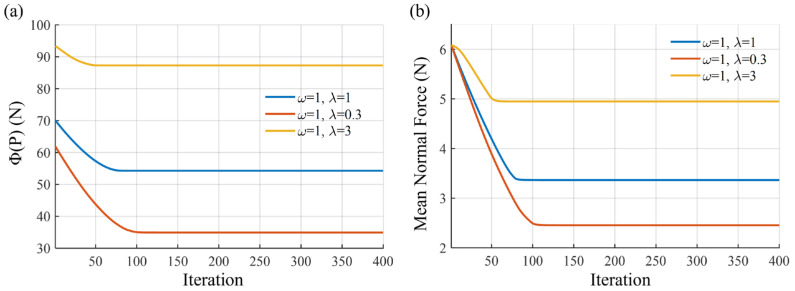
Iterative process: (**a**) Convergence of the objective function under different *ω*/*λ* ratios; (**b**) the mean normal force under different *ω*/*λ* ratios.

In this work, diagonal weighting matrices $W_{p}=\omega I$ and $W_{i}=\lambda I$ where ω and *λ* are positive weighting factors. Convergence curves of the objective function under different ω/*λ* are provided in Figure 2a, which shows that decreasing *λ* leads to a lower converged value of the objective function *Φ*(*P*). This indicates that the optimization allows the descriptive matrix *P* to approach singularity to a greater extent, thereby relaxing the penalty on tangential force components. The corresponding physical interpretation is revealed in Figure 2b, where the *F*_mean_ of all contact units decreases monotonically with *λ*. This confirms that a smaller *λ* results in reduced average interface pressure, at the cost of operating closer to the friction constraint boundary. Taken together, Figure 2 demonstrates a clear and consistent trade-off mechanism between pressure suppression and distribution safety: a larger *λ* enforces a more conservative solution with higher normal forces and increased robustness against slip, whereas a smaller *λ* favors lower interface pressure while tolerating reduced margins to the friction limit.

**Figure 3:**
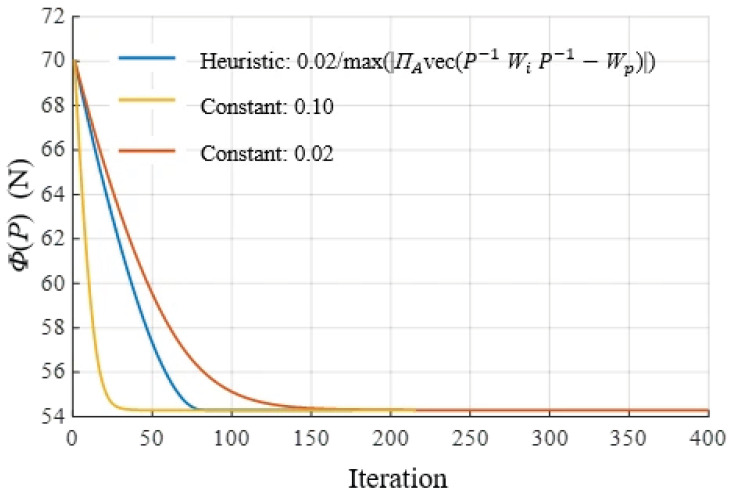
Comparison of convergence behavior under different α_k_ (ω = λ = 1).

Based on this objective function, the linear constrained gradient flow method is employed to compute the exponentially convergent solution of the descriptive matrix, thereby achieving pressure optimization. The gradient flow equation for the objective function $\Phi \left(P\right)$ is

(6) $$\mathrm{vec}\left(\dot{P}\right)=-\nabla \Phi \left(P\right)=\Pi_{A}\mathrm{vec}\left(P^{-1}W_{i}P^{-1}-W_{p}\right)$$

where $\Pi_{A}=I-A^{+}A$ is the linear projection operator, and the constraint matrix *A* is formed by both the force equilibrium constraints and structural constraints describing the matrix. The gradient flow is discretized using the Euler integration method as follows

(7) $$\mathrm{vec}\left(P_{k+1}\right)=\mathrm{vec}\left(P_{k}\right)+\alpha_{k}\Pi_{A}\mathrm{vec}\left(P^{-1}W_{i}P^{-1}-W_{p}\right)$$

where *α_k_* denotes the step size, which must be chosen to ensure convergence of the objective function. Both constant step sizes and a heuristic adaptive step size

$$\alpha_{k}=\frac{0.02}{\max\left(\left|\;\Pi_{A}\mathrm{vec}\left(P^{-1}W_{i}P^{-1}-W_{p}\right)\right|\right)}$$

are investigated. Figure 3 illustrates the influence of the step size α on the convergence characteristics of the algorithm, with ω = λ = 1. It can be observed that, while all tested step size strategies converge to the same equilibrium point, larger constant step sizes significantly accelerate convergence. The heuristic step size achieves faster convergence in the early stages but may exhibit mild oscillations near the equilibrium, which is a typical characteristic of adaptive step strategies. The algorithm commences from an initial force distribution satisfying the constraints and sets a convergence error for iteration. The resulting convergent solution yields the optimal force distribution for each unit.

## 3. Optimization of the Spatial Layout of Socket Contact Units

To optimize the comfort and load-bearing performance of the prosthetic socket, this section determines the spatial layout of the contact units from two perspectives. Firstly, biomechanical experiments are conducted to identify the load-tolerant regions of the forearm soft tissues, which are selected as the primary load-bearing areas to achieve anatomically informed load distribution optimization and improve biomechanical compatibility. Secondly, based on a multi-contact unit force optimization algorithm, the mechanical performance of different layout schemes is evaluated under various motion patterns, leading to the selection of the unit layout with the optimal mechanical properties. Biomechanical experiments are first carried out to investigate the distribution characteristics and biomechanical patterns of the pressure pain thresholds in the forearm soft tissues. In practical application, individualized adaptation should be performed by integrating the underlying universal principles with the specific anatomical and physiological characteristics of the residual limb.

### 3.1. Biomechanical Experiments

Ideally, biomechanical experiments on the forearm should recruit forearm amputees with varying stump conditions as participants. However, due to the low proportion of this population, it is challenging to assemble a sample group with broad representativeness and reference value. Therefore, this experiment enrolled 10 healthy participants (gender: 5 female/5 male; age: 23.9 ± 1.0 years. Detailed data is shown in Table 1) in good physical condition and free from skin diseases. Prior to the experiment, subjects were thoroughly informed of the experimental purpose, procedures, and potential risks.

**Figure 4:**
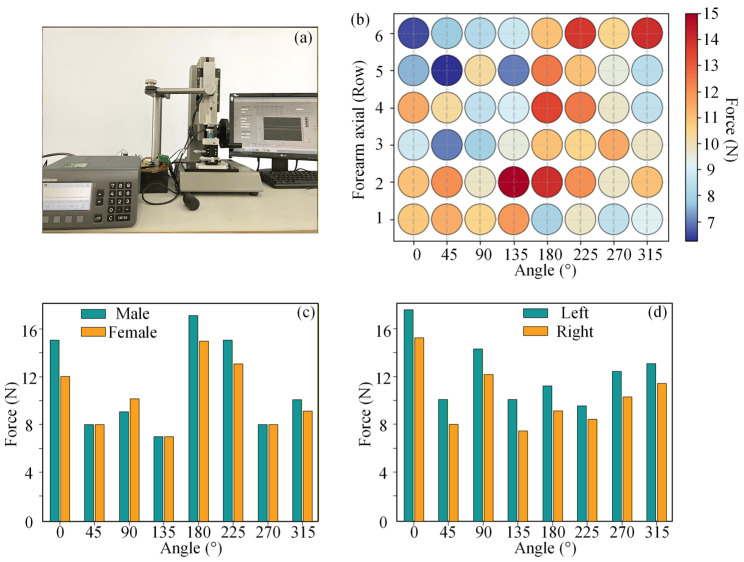
Biomechanics experiment: (**a**) Experimental platform; (**b**) location of pain threshold measurement points and distribution of pain thresholds; (**c**) gender differences in pressure pain threshold distribution; (**d**) bilateral forearm pressure pain threshold distribution.

Pressure pain threshold (PPT) was defined as the ratio of the interface pressure at which the subject first perceives pain to the area of the indenter [32]. An indenter (diameter = 4 mm) was used to apply load perpendicular to the skin surface at a rate of 1 mm/s until the subject reports pain. The edges of the indenter were rounded to avoid pain caused by stress concentration. The experimental setup, shown in Figure 4a, includes a manually driven loading platform and a six-axis force sensor (Nano 17). Taking the right arm as an example, the midpoint of the elbow crease was set as the coordinate origin. The positive longitudinal axis extends from the origin toward the midpoint of the wrist crease, and the positive transverse axis extends from the origin toward the radial side. The forearm is divided axially into seven equal segments, excluding the two ends (elbow line and wrist line), yielding six rows in total. Measurement points were selected at 45° intervals in a counter-clockwise direction (viewed from the wrist toward the elbow), with eight points per row. The measurement point matrix is illustrated in Figure 4b. Each point was measured three times at five-minute intervals, and the mean value was adopted as the PPT for that point. The PPT for all points on the forearm constituted a PPT distribution matrix [33].

Since force, displacement, forearm length, and circumference are continuous variables, their measured values exhibit consistent variation within equidistant intervals, while gender and handedness are dichotomous variables. Pearson correlation coefficient was used to assess linear relationships between continuous variables. Point-biserial correlation (a special case of Pearson correlation) was employed for relationships between dichotomous variables and continuous variables. The results showed that correlation coefficients between PPT and forearm length, circumference, gender, and handedness were 0.13, 0.08, −0.1 and −0.05, respectively (positive/negative values denote positive/negative correlations). This demonstrates that morphological parameters show no significant association with the pain threshold. Thus, for socket design, inter-individual variation can be considered relatively small, and load-bearing area distribution tends to be consistent, allowing for fine-tuning based on the specific tissue properties of the patient. *p*-values for the male and female pressure pain threshold matrices were 0.61 and 0.65, respectively, indicating no statistically significant gender-related differences in pain distribution. The average PPT for females was approximately three-quarters that of males. This implies that design for females may present greater challenges.

The load-bearing capacity distribution diagram shown in Figure 4b indicates that the majority of the proximal forearm region exhibits high load-bearing capacity. Sensitive areas should be avoided for unit placement and accommodated via recessed reliefs on the inner socket wall. Based on the results of the biomechanical experiment shown in Figure 4c,d, the following guiding principles for socket unit design and distribution can be derived: Gender exerts no significant influence on the spatial distribution of pain thresholds, although the average threshold is lower in females. The mechanical deformation characteristics of the left and right forearms are similar, allowing for consistent socket unit distribution designs.

### 3.2. Spatial Layout of Multi-Contact Units

The spatial arrangement of multi-contact units plays a decisive role in determining the overall mechanical performance and comfort of the prosthetic socket [34]. The prosthesis coordinate system is defined as follows: the origin is set at the center of mass of the prosthetic system; the *x*-axis is normal to the palmar plane and directed anteriorly, the *y*-axis coincides with the forearm longitudinal axis and points from the wrist toward the elbow, and the *z*-axis lies in the palmar plane and points toward the radial side.

To effectively resist tilting and pitching moments, the load-bearing units are distributed across two axial layers: proximal and distal. The selection of these layer positions must balance interface stability with the need to avoid mechanically sensitive areas, such as the distal end of the residual limb, as well as the cubital fossa and antecubital region, which could impede elbow mobility. A safety margin of 2–3 cm should be maintained between the unit edges and both the elbow line and the residual limb end. Biomechanical experiments indicate that the soft tissues in the proximal forearm region exhibit high load tolerance. Therefore, a cross-section 5 cm distal to the elbow line (*y* = 5 cm) is selected as the proximal bearing layer. The distal bearing layer position is determined individually based on residual limb length and PPT distribution; for this study, the section at *y* = 15 cm is used for analysis.

**Figure 5:**
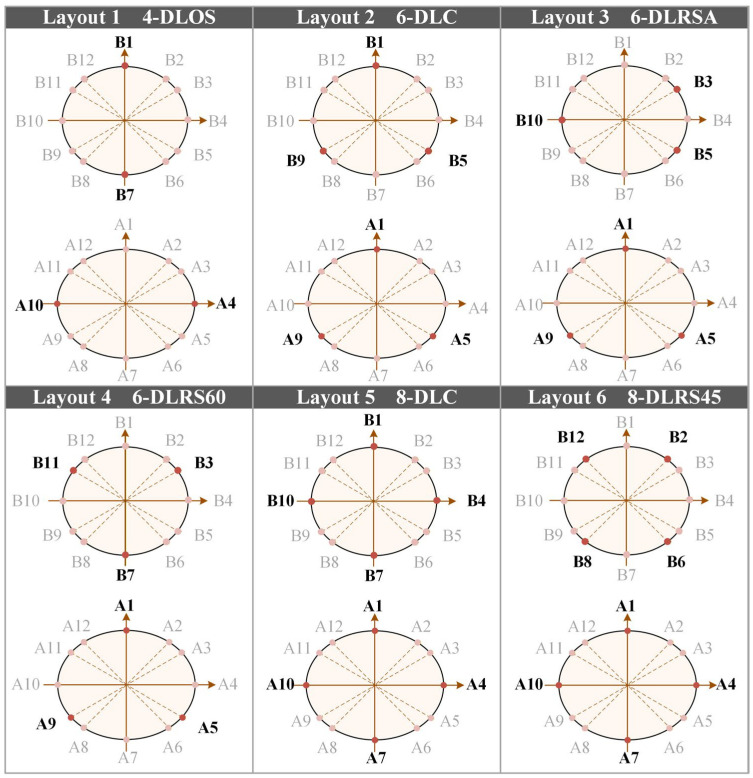
Schematic of socket unit layout. Units located at the cross-section *y* **=** 50 mm are labeled as A, while those located at *y* = 150 mm are labeled as B.

To investigate the effects of key parameters, including the number of units, circumferential symmetry, and radial stagger angle on system performance, six distinct layout schemes were designed, as shown in Figure 5. These schemes range from basic to highly redundant configurations [34] and include a 4-unit double-layer orthogonally symmetric layout (4-DLOS), a 6-unit double-layer coincident layout (6-DLC), a 6-unit double-layer radially staggered asymmetric layout (6-DLRSA), a 6-unit double-layer radially staggered 60° layout (6-DLRS60), an 8-unit double-layer coincident layout (8-DLC), and an 8-unit double-layer radially staggered 45° layout (8-DLRS45). As illustrated in Figure 5, Section A (proximal plane) is located at *y* = 50 mm, and Section B (distal plane) is at *y* = 150 mm. Using the midpoint of the elbow line as the circumferential reference origin (0°), unit positions are numbered A1–A12 and B1–B12, corresponding to angles of 0°, 45°, 60°, 90°, 120°, 135°, 180°, 225°, 240°, 270°, 300°, and 315°, respectively. Taking the 6-unit double-layer radially staggered 60° layout as an example, the three units on proximal plane A are evenly spaced 120° apart circumferentially, while the three units on distal plane B are rotated 60° relative to those on plane A, forming a staggered structure.

To evaluate the adaptability of the socket under different task scenarios, four representative biomechanical task scenarios are selected, which cover common loading conditions such as axial force, pure torque, and composite loads. These scenarios effectively reflect the complex mechanical conditions encountered in practical socket applications:

(1) Suspension Mode (Axial load) simulates a pure axial loading condition, such as carrying an external weight with the prosthetic hand. In this mode, only the axial force component along the forearm longitudinal axis is applied;

(2) Rotational Mode (Torsional load) represents a pure torsional loading condition around the forearm longitudinal axis, corresponding to tasks such as twisting a bottle cap or a key. In this case, only the torsional moment component is applied;

(3) Forearm Elevation Mode simulates the pitching motion of the forearm during arm elevation while holding an object. This mode includes a force component normal to the palmar plane and a pitching moment within the palmar plane, reflecting the combined effects of gravity and joint rotation during elevation;

(4) Combined Load Mode represents a complex multi-directional loading condition commonly encountered in daily activities, such as operating a door handle. Simultaneous force and moment components are applied along and about all coordinate axes to comprehensively evaluate the robustness and load distribution capability of the socket under realistic operating conditions.

To quantitatively evaluate the biomechanical performance of different layouts, three key metrics were defined based on the normal contact forces generated by the socket units:

(1) Peak normal force (*F*_max_): the maximum normal contact force among all units, which reflects the risk of localized high loading at the human–socket interface. Minimizing *F*_max_ helps reduce discomfort and the potential for soft tissue injury.

(2) Mean normal force (*F*_mean_): the average normal contact force across all units, representing the overall load intensity applied to the residual limb. Lower *F*_mean_ is conducive to improved long-term wearing comfort.

(3) Force distribution variance (Var(*F*)): the variance of normal contact forces among all units, which characterizes the uniformity of load distribution at the interface. A lower variance indicates more balanced force sharing and improved stability under multi-directional loading conditions.

Together, these three metrics form a quantitative basis for evaluating interface comfort and mechanical stability.

**Figure 6:**
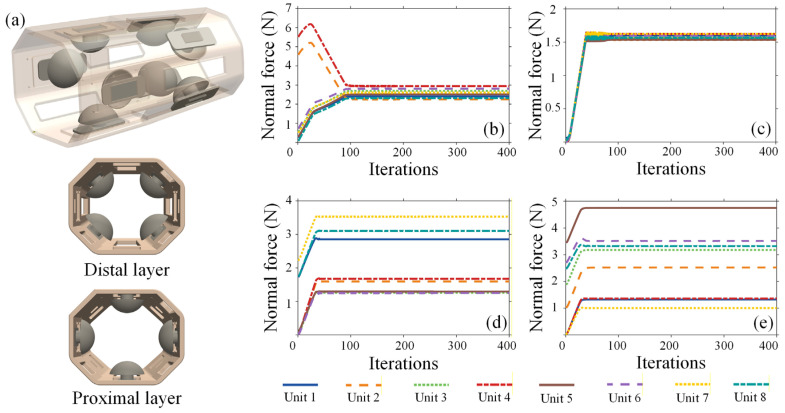
Socket with an 8-unit double-layer symmetrical radial staggered array. (**a**) Unit spatial configuration: It consists of a shell body and eight contact units, each with a hemispherical interface to distribute contact stress. Four proximal units are placed at 0°, 90°, 180°, and 270°; four distal units are radially staggered by 45°; (**b**) normal forces under suspension mode; (**c**) normal forces under rotational load; (**d**) normal forces under forearm elevation mode; (**e**) normal forces under combined load.

**Figure 7:**
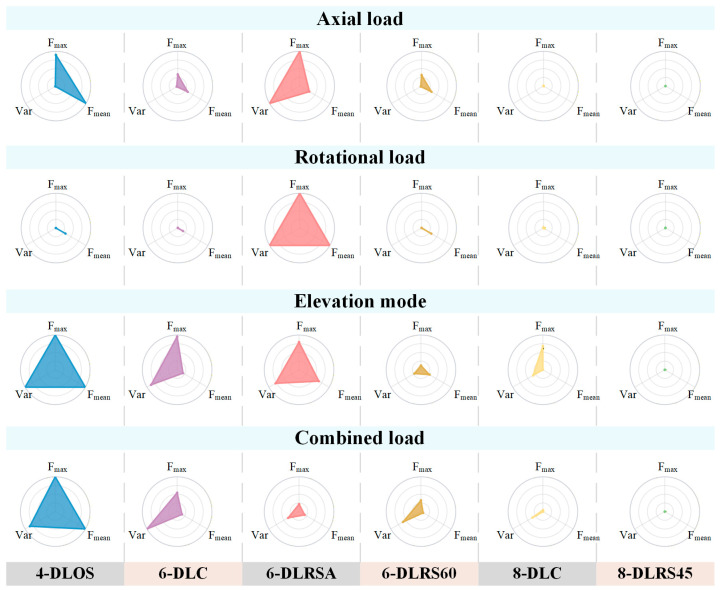
Faceted radar charts of comprehensive mechanical performance for different layout schemes.

Based on a dynamic force optimization algorithm, the force distribution of six configurations in representative task scenarios is calculated. Taking 8-DLRS45 as an example, the structure of the socket based on this layout is shown in Figure 6a. The socket consists of a shell body and eight contact units, each with a hemispherical interface to distribute contact stress. Four proximal units are placed at 0°, 90°, 180°, and 270°; four distal units are radially staggered by 45°. The normal force distribution of its individual units under the four task scenarios is shown in Figure 6b–e. A systematic quantitative evaluation of all six layouts was performed using three biomechanical metrics. The results are summarized in Table 2. To facilitate an intuitive comparison of the comprehensive mechanical performance of different layout schemes, the normalized values of *F*_max_, *F*_mean_, and Var(*F*) are visualized using faceted radar charts, as shown in Figure 7. A larger enclosed area indicates inferior comprehensive performance, whereas a more compact polygon reflects lower interface pressure and improved load distribution uniformity. This visualization enables a direct assessment of the trade-offs among different comfort-related metrics across task scenarios and highlights the relative advantages of each layout.

The technique for order preference by similarity to ideal solution (TOPSIS) was employed to evaluate the mechanical performance of six layouts under four representative task scenarios. Weights of 0.4, 0.3, and 0.3 were assigned to *F*_max_, *F*_mean_, and Var(*F*), respectively. The positive ideal separation measure *S*^+^, negative ideal separation measure *S*^−^ and relative closeness coefficient *C_i_* of each layout to the ideal solution are as shown in Table 3. Results indicate that 6-DLRSA performed poorly in most modes, with its high distribution variance highlighting the inadequacy of asymmetric layouts in achieving load uniformity. 6-DLRS60 was optimal under torque conditions, demonstrating that performance is not simply proportional to the number of units. 8-DLC ranked second overall after 8-DLRS45, with their performance difference mainly attributable to the inter-layer stagger angle.

### 3.3. Discussion

This study aims to enhance the mechanical transmission efficiency and biomechanical compatibility of upper-limb prosthetic sockets by integrating a multi-unit active structure with task-oriented dynamic force optimization. Biomechanical experiments indicate that most regions of the proximal forearm exhibit relatively high load-bearing capacity, whereas mechanically sensitive areas should be relieved from sustained contact. No significant influence of gender or handedness on the spatial distribution of pressure pain thresholds was observed, although females generally exhibited lower absolute thresholds. These findings provide a physiological basis for selectively placing load-bearing units while avoiding sensitive regions in socket design. A limitation of this study is the relatively small sample size of the biomechanical experiments, and the fact that all subjects were healthy individuals whose soft tissue characteristics differ from those of amputees. Therefore, in practical applications, personalized fitting must be achieved by integrating the common principles revealed in this paper with the specific anatomical structure and physiological characteristics of the individual residual limb.

In addition to the physiological observations, the quantitative mechanical results presented in Table 2 provide direct evidence supporting the proposed socket design strategy. Across all evaluated task scenarios, increasing the number of contact units generally reduced *F*_max_ and *F*_mean_, indicating improved load-sharing capability. However, layouts lacking circumferential symmetry, such as the 6-DLRSA configuration, exhibited markedly higher Var(F), particularly under combined loading conditions, despite comparable average force levels. The results demonstrate that unit number alone is insufficient to ensure interface comfort and safety. Instead, spatial symmetry and inter-layer coordination play a decisive role in suppressing localized pressure concentration and maintaining stable force redistribution under multi-directional loads.

Building upon the above biomechanical findings, the proposed multi-unit active socket design provides a systematic solution to several critical challenges commonly encountered in conventional full-contact sockets, including inadequate heat dissipation, excessive pressure on sensitive areas, stress concentration, and complex adaptation processes. Structurally, the multi-unit configuration intentionally reduces continuous contact between the socket and the residual limb by introducing discrete load-bearing units separated by open regions. This design facilitates air circulation at the socket–skin interface, thereby mitigating heat accumulation during prolonged wear.

From a biomechanical perspective, pressure on sensitive areas is alleviated through the selective placement of contact units in load-tolerant regions of the forearm, as identified by the pressure pain threshold experiments presented in Section 3.1. Mechanically sensitive regions are accommodated by recessed or non-contact areas of the socket frame, ensuring that sustained normal pressure is avoided in these zones. Stress concentration is further mitigated through the combined use of hemispherical contact interfaces and the task-oriented dynamic force optimization algorithm, which continuously redistributes normal forces among multiple units under varying external loads to suppress localized pressure peaks and enhance overall pressure uniformity.

Furthermore, unlike traditional sockets that rely heavily on precise geometric congruence between the socket and residual limb, the proposed design achieves adaptability primarily through active force regulation. By dynamically adjusting the output forces of individual units in response to task-specific loading conditions, inter-individual anatomical variability, and long-term physiological changes of the residual limb, the system reduces the complexity associated with socket fitting and re-adjustment. Consequently, the multi-unit active socket shifts the adaptation paradigm from static shape matching toward dynamic, task-oriented pressure control, offering improved comfort, safety, and robustness in multi-task usage scenarios.

A comparative analysis of the six layouts further reveals why the eight-unit double-layer radially staggered configuration outperforms the others. Under simple axial or pure torsional loads, several layouts achieve similar performance. However, under forearm elevation and combined loading conditions, which more closely reflect real-world prosthetic use, the 8-DLRS45 layout consistently exhibits the lowest *F*_max_ and the smallest force variance. This indicates superior robustness against load coupling and task variability. In contrast, coincident double-layer layouts tend to form overlapping stress paths, leading to higher sensitivity to load direction changes. The staggered inter-layer arrangement effectively mitigates stress superposition, providing mechanical redundancy that enhances adaptability across diverse task scenarios: under extreme or composite loading conditions, all units can be engaged synergistically to ensure stable suspension, whereas under simpler motion modes, task-appropriate subsets of units can be selectively activated according to their mechanical advantage.

From a practical implementation perspective, this layout is well suited to realization within a modular active socket architecture. Each contact unit can be implemented as an independently actuated module integrating a rigid structural base, a compliant hemispherical interface for stress dispersion, and a local force-regulation mechanism. These units can be mounted onto the socket shell structure using standardized inter-faces, enabling precise placement consistent with the optimized circumferential angles and axial layer positions.

In practical operation, external loads acting on the prosthetic system during daily activities can be obtained or estimated from prosthetic system sensing or control signals and used as inputs to the proposed dynamic force optimization algorithm. The resulting optimal normal force distribution is then tracked by local closed-loop controllers within each unit, enabling accurate and stable regulation of interface pressure during dynamic tasks. Furthermore, the selective activation strategy enabled by the eight-unit layout offers additional benefits for long-term wearing comfort and safety. Rather than continuously driving all units, dynamically switching between mechanically equivalent unit combinations allows periodic redistribution of load-bearing regions. This mechanism helps promote soft tissue blood circulation and mitigate the risk of localized pressure accumulation during prolonged use. Even in task scenarios where this layout is not theoretically optimal for a single loading mode (e.g., pure torsion), the controller can prioritize the activation of the most mechanically advantageous unit subset.

In this context, passive adaptation refers to conventional prosthetic sockets with fixed geometry and static force distributions, in which interface forces are primarily determined during fabrication or fitting and remain unchanged during daily use. Such sockets rely mainly on passive material compliance or liner deformation to accommodate variations in residual limb morphology and external loads, which limits their ability to prevent localized pressure accumulation during complex or long-duration activities. By contrast, active care in the proposed system is achieved through the synergistic integration of redundant multi-contact units and a task-oriented dynamic force optimization strategy. The socket actively adjusts the output force of each contact unit in real time according to external loads and motion patterns, while enabling dynamic pressure redistribution through selective unit activation. Consequently, the proposed layout not only demonstrates superior static mechanical performance but also provides a practical and scalable platform for intelligent prosthetic sockets capable of evolving from passive load accommodation toward proactive, long-term biomechanical support.

## 4. Conclusions

In this study, an active prosthetic socket design strategy based on intelligent physiological adaptation is proposed. Specifically, a task-oriented dynamic force optimization algorithm for multiple contact units is developed to dynamically regulate the output force of each unit under varying external loads, thereby achieving stable suspension with minimized interface pressure. Biomechanical experiments were performed to provide a physiological rationale for the contact unit layout. A multi-objective evaluation framework centered on comfort and stability was established, which employs *F*_max_, *F*_mean_, and Var(*F*) as evaluation metrics to compare the mechanical performance of six layout schemes under four representative task scenarios. The results indicate that the 8-DLRS45 exhibits stable and superior mechanical performance across diverse task scenarios, especially under combined load conditions, thus attaining a global optimal equilibrium state.

This study improves the dynamic adaptability of prosthetic sockets from the perspectives of mechanical transmission efficiency and biomechanical compatibility, thereby providing a novel theoretical framework and design paradigm for the development of high-performance upper limb prosthetic sockets in the future. Nevertheless, the mechanical performance evaluation of the optimal spatial configuration currently relies solely on theoretical analysis and calculations. Subsequent comparative experimental validation with conventional sockets is therefore warranted to further verify the practical feasibility and superiority of the proposed design. Future work will include finite element simulations and prototype-based experimental validation to further corroborate the theoretical findings presented in this study.

## Figures and Tables

**Table 1:** Characteristics of participants involved in the biomechanical experiment.

Participant	Sex	Age (Years)	Height (cm)	Mass (kg)	Forearm Length (cm)	Forearm Circumference (cm)
1	Male	24	170	72	26	21.5
2	Male	23	173	66	27	19.5
3	Male	24	177	84	26	22
4	Male	25	184	95	28	24.3
5	Male	24	170	58	24.5	19.5
6	Female	23	168	57	23.5	18.2
7	Female	24	166	55	24.5	18.8
8	Female	23	160	52	23	17.8
9	Female	26	161	49	24	18.3
10	Female	23	160	52	24	18.5
Mean ± SD	-	23.9 ± 0.94	168.9 ± 7.37	64.1 ± 14.66	25.05 ± 1.54	19.84 ± 1.99

**Table 2:** Biomechanical performance metrics under representative task scenarios.

Layout Characteristics	*F*_max_ (N)	*F*_mean_ (N)	Var(F) (N^2^)
Axial load *F_ext_* = [0, 5, 0, 0, 0, 0]^T^			
4-DLOS	4.2927	4.0804	0.0249
6-DLC	3.3028	3.0587	0.0320
6-DLRSA	4.4855	3.0517	0.4625
6-DLRS60	3.2705	3.0578	0.0282
8-DLC	2.7273	2.5737	0.0152
8-DLRS45	2.7168	2.5681	0.0157
Rotational load *F_ext_* = [0, 0, 0, 0, 5, 0]^T^			
4-DLOS	1.5928	1.5870	0.00005
6-DLC	1.5966	1.5827	0.00009
6-DLRSA	2.2727	1.6074	0.10240
6-DLRS60	1.5952	1.5871	0.00004
8-DLC	1.6073	1.5783	0.00053
8-DLRS45	1.596	1.5777	0.00026
Elevation mode *F_ext_* = [2, 0, 0, 0, 0, 0.5]^T^			
4-DLOS	5.0978	3.2963	1.8895
6-DLC	5.019	2.3072	1.7357
6-DLRSA	4.7753	2.8720	1.6398
6-DLRS60	3.721	2.4077	1.0107
8-DLC	4.5996	2.0687	1.1304
8-DLRS45	3.5313	2.0706	0.7682
Combined load *F_ext_* = [2, 2, 2, 0.5, 0.5, 0.5]^T^			
4-DLOS	7.6949	6.2091	2.8832
6-DLC	6.3365	3.1982	3.1408
6-DLRSA	5.3707	3.2907	2.0924
6-DLRS60	5.689	2.8791	2.4717
8-DLC	4.871	2.6381	2.0643
8-DLRS45	4.7467	2.6202	1.5032

**Table 3:** Performance evaluation of the layout schemes.

Rank	*S* ^+^	*S* ^−^	*C_i_*	No.
Axial load *F_ext_* = [0, 5, 0, 0, 0, 0]^T^				
1	0.0011	0.3247	0.9966	8-DLRS45
2	0.0014	0.3247	0.9957	8-DLC
3	0.0263	0.2998	0.9194	6-DLRS60
4	0.0298	0.2962	0.9086	6-DLC
5	0.0728	0.2523	0.7761	4-DLOS
6	0.2881	0.0367	0.1130	6-DLRSA
Rotational load *F_ext_* = [0, 0, 0, 0, 5, 0]^T^				
1	0.0013	0.2999	0.9957	6-DLRS60
2	0.0015	0.2999	0.9950	4-DLOS
3	0.0026	0.2998	0.9914	6-DLC
4	0.0076	0.2996	0.9752	8-DLRS45
5	0.0155	0.2995	0.9508	8-DLC
6	0.2999	0.0000	0.0000	6-DLRSA
Elevation mode *F_ext_* = [2, 0, 0, 0, 0, 0.5]^T^				
1	0.0000	0.1222	1.0000	8-DLRS45
2	0.0529	0.0825	0.6093	6-DLRS60
3	0.0595	0.0759	0.5606	8-DLC
4	0.0789	0.0565	0.4174	6-DLRSA
5	0.0839	0.0515	0.3804	6-DLC
6	0.1222	0.0000	0.0000	4-DLOS
Combined load *F_ext_* = [2, 2, 2, 0.5, 0.5, 0.5]^T^				
1	0.0000	0.2241	1.0000	8-DLRS45
2	0.0270	0.1976	0.8798	8-DLC
3	0.0483	0.1766	0.7852	6-DLRSA
4	0.0548	0.1702	0.7564	6-DLRS60
5	0.0783	0.1467	0.6521	6-DLC
6	0.2241	0.0000	0.0000	4-DLOS

## Data Availability

Data are contained within this article.

## Abbreviations

The following abbreviations are used in this manuscript:

PPT	Pressure Pain Threshold
TOPSIS	Technique for Order Preference by Similarity to Ideal Solution

## Nomenclature

Symbol	Description
*f_i_*	Contact force vector applied by the *i*-th unit of the socket
*f_i,n_*	Normal force perpendicular to residual limb skin surface
*f_i,t_*_1_	First tangential force component of the *i*-th unit
*f_i,t_*_2_	Second tangential force component of the *i*-th unit
*μ_i_*	Equivalent friction coefficient of the *i*-th unit of the socket
*F*_max_	Maximum normal contact force among all socket units
*F*_mean_	Mean normal contact force across all socket units
Var(F)	Variance of normal contact forces among socket units
*G*	Force transmission matrix of the socket system
*G_i_*	Force transmission matrix of the *i*-th unit
*F_unit_*	System force vector composed of the output forces from all units
*W_ext_*	External load vector
*P_i_*	Descriptive matrix of the *i*-th contact unit
*P*	Global block-diagonal descriptive matrix of all units
*Φ(P)*	Objective function of the pressure optimization problem
*W_p_*	Weighting matrix for force suppression
*W_i_*	Weighting matrix for force distribution uniformity
*Π_A_*	Linear projection operator associated with constraints
*α_k_*	Step size at the *k*-th iteration of the gradient flow
*i*	Index of contact unit
*k*	Iteration index
